# Genetic Diversity of *Theileria parva* and *Anaplasma* spp. Isolated From Ticks Collected From Kiambu County, Kenya

**DOI:** 10.1155/bmri/5577637

**Published:** 2026-05-13

**Authors:** Peter Gichuki, Caroline Wasonga, Christine Adhiambo, Joel Lutomiah

**Affiliations:** ^1^ Department of Biochemistry, University of Nairobi, Nairobi, Kenya, uonbi.ac.ke; ^2^ Centre for Virus Research, Kenya Medical Research Institute, Nairobi, Kenya, kemri.org

**Keywords:** *Anaplasma* spp., Kiambu County, *Theileria parva*, tick-borne diseases (TBDs)

## Abstract

Environmental changes and human activities such as deforestation and expansion of agricultural land are increasing tick‐borne diseases including Anaplasmosis, Babesiosis, Ehrlichiosis, and Theileriosis. These diseases, which affect animals, can be transmitted to humans through tick bites. Kiambu County′s warm, wet climate provides an environment conducive to tick breeding and development. This study investigated the circulation and genetic diversity of *Theileria parva* and *Anaplasma* spp. in tick samples collected from Kiambu County, Kenya. Ticks were collected from cattle, goats, and sheep using animal grooming methods and morphologically identified and organized into 129 pools. Total DNA was extracted from tick pools using the sodium dodecyl sulfate extraction method. The 18S rRNA hypervariable region was amplified for *T. parva* detection, and the 16S rRNA gene was used for *Anaplasma* spp. detection. A total of 716 ticks were collected, with *Rhipicephalus evertsi evertsi* (*n* = 585, 81.7%) being the most abundant species. Molecular analysis indicated the presence of *T. parva* in *n* = 8 pools of *Rh. e. evertsi. Anaplasma* species was detected in three pools of *Rh. e. evertsi*, one pool of *A. variegatum*, and one pool of *H. truncatum*. Phylogenetic analysis revealed that eight *T. parva* samples clustered closely with isolates from Uganda and Mexico, suggesting potential historical or ecological links between regional isolates and international strains, although direct transmission cannot be confirmed. For *Anaplasma* spp., phylogenetic analysis identified *Anaplasma ovis* and *Anaplasma bovis* in ticks collected from cattle and sheep, including *Rh. e. evertsi*, *A. Variegatum*, and *H. truncatum*, with single nucleotide polymorphisms (SNPs) identified within the *Anaplasma* sequences. The findings emphasize the importance of continued molecular surveillance of tick‐borne pathogens, characterization, and the development of targeted tick control measures to mitigate the impact of tick‐borne diseases in livestock.

## 1. Introduction

Livestock farming is a key economic activity in Kenya, supporting 39% of households [1] and contributing to 12% of the national Gross Domestic Product (GDP) and 50% of agricultural GDP [2]. The measure of agricultural GDP only considers the livestock products sold in the open market, whereas cattle, goats, and sheep are critical for food security, income, and social capital [3]. For instance, livestock provide milk and meat, supply hides, manure, fuel, and traction power for pulling agricultural machinery [4, 5], which signifies the essential roles that livestock play in people′s livelihoods.

Environmental changes and human activities, such as deforestation and agricultural expansion, disrupt ecosystems, increasing contact between ticks, wildlife, and livestock, and raising the risk of tick‐borne diseases like Anaplasmosis, Babesiosis, Ehrlichiosis, and Theileriosis [6]. Kiambu County′s warm climate and heavy rainfall create ideal conditions for ticks, which transmit pathogens to animals and humans [7]. Ticks belonging to the genera *Amblyomma, Dermacentor, Hyalomma, Haemaphysalis*, and *Rhipicephalus* are medically important since they are hosts for various mammalian pathogens, some of which affect humans [8, 9].

*Theileriosis* (East Coast fever), caused by *Theileria parva*, is endemic in Kenya and is transmitted by *Rhipicephalus appendiculatus*. The *T. parva* genome is approximately 8.3 Mb and consists of four chromosomes [10, 11]. The average genome‐wide recombination rate for *T. parva* is relatively high at 0.22 cM Kb−1 per meiotic generation, indicating that analysis of relatively few progeny can provide high mapping resolution [11]. The disease causes lymph node enlargement, anemia, and mortality in cattle, with high prevalence reported in western Kenya [12, 13]. Seven distinct *Theileria* species have been identified in Ngong in Kajiado County in the Rift Valley region and in Machakos County in the eastern region of Kenya [14].

Anaplasmosis, caused by *Anaplasma* spp. including *Anaplasma marginale* and *Anaplasma ovis*, threatens livestock productivity. *Anaplasma* is an obligate intracellular bacterium with a small genome size ranging from 1 to 1.5 megabase pairs [15, 16]. The 16S ribosomal RNA gene within the bacterial genome has a slow evolution rate, which makes it an appropriate tool for the identification of species and phylogenetic analysis [17]. In Kenya, *A. marginale, A. bovis*, and *A. platys* have been detected in cattle in periurban Nairobi [18] and Lambwe Valley [19]. In the Lambwe Valley wildlife‐livestock interface, *Anaplasma* species accounted for 78.9% herd prevalence and 45.7% of animal‐level prevalence. Among the sampled herds, the prevalence of *A. bovis* was 57.9%, *A*. *platys* was 51.6%, whereas *A. marginale* accounted for 4.2% herd prevalence [19]. Clinical signs in cattle include anemia, fever, and death [20], whereas human infections, though rare, can lead to severe complications including respiratory failure, bleeding problems, organ failure, and death [21].

We conducted a cross‐sectional study in Kiambu County to determine the diversity of tick species collected from livestock, and the prevalence and genetic diversity of *T. parva* and *Anaplasma* spp. in the ticks in order to inform targeted disease control.

## 2. Materials and Methods

### 2.1. Study Sites

**Figure 1:**
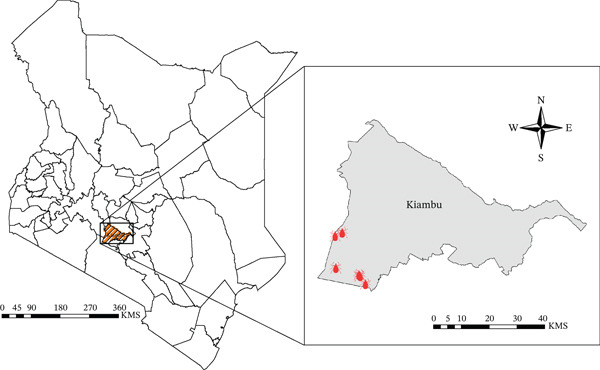
Image showing the various sample collection points within Kiambu County, Kenya. The sites selected for this study included Gatune 66, Osero 21, Nachu, Riu Nderi, Karai Bomboini, Karera 6, and Karera 8.

This study was conducted in Kiambu County, Kenya, a region located in the central highlands between latitudes 0°25 ^′^ and 10°20 ^′^ south and longitudes 36°31 ^′^ and 37°15 ^′^ east. Kiambu County′s warm and temperate climate, characterized by annual rainfall ranging from 600 to 2000 mm, varied elevation (1200–2550 m above sea level), and a mosaic of periurban and rural land use, creates a favorable environment that influences tick vector ecology and host‐pathogen dynamics [22]. Seven georeferenced sites were selected for investigation: Gatune 66, Osero 21, Nachu, Riu Nderi, Karai Bomboini, Karera 6, and Karera 8 (Figure 1).

### 2.2. Tick Collection

Ticks were collected once in each study site from the skin of infested domesticated animals (cattle, sheep, and goats) in Kiambu County, Kenya (Figure 1) between October 2019 and December 2020. A targeted sampling strategy was employed, where animals were selected for inclusion based on the presence of visible tick infestations upon physical examination. Visibly infested animals were restrained, and ticks were carefully removed from the skin using blunt forceps and placed in labelled sterile centrifuge tubes. Tick samples were transported in liquid nitrogen to the arbovirus and viral hemorrhagic fevers (VHF) laboratory at Kenya Medical Research Institute (KEMRI). They were morphologically identified to species using appropriate taxonomic keys and pooled (≤ 8 ticks per pool) by sex, species, developmental stage, animal host (cattle, sheep, and goats), site, and date of collection using the Taxonomy of African Ticks: An Identification Manual [23] to minimize variability and increase polymerase chain reaction (PCR) throughput while reducing resource consumption. Pooling allowed for efficient screening without compromising detection sensitivity for the pathogens.

Statistical analysis was performed using Microsoft Excel to determine tick species distribution and diversity. The Shannon Diversity Index (H ^′^) was used to measure the diversity of tick species, accounting for both abundance and evenness using the formula: *H* ^′^ = –*Σ* (*p*_*i*_ × ln *p*_*i*_), where *p*_*i*_is the proportion of individuals belonging to the *ith* species [24]. Pielou′s evenness (J ^′^) was subsequently used to assess how uniformly individuals are distributed across species using the formula: J ^′^ = *H* ^′^/ln(*S*), where *S* is the total number of tick species (species richness) [25]. Higher values of H ^′^ and J ^′^ indicate greater diversity and more even distribution, respectively.

### 2.3. Tick Homogenization, DNA Extraction, and Molecular Analysis

Each tick pool was homogenized in a prechilled mortar with alundum sand and 2 mL homogenization media, consisting of Minimum Essential Media (MEM) (Sigma‐Aldrich) supplemented with 2% antibiotic and antimycotic (fungizone (1 *μ*L/mL, penicillin (100 *μ*g/mL), streptomycin (100 *μ*g/mL)), 2% L‐glutamine, and 15% heat‐inactivated fetal bovine serum (FBS) [26]. Homogenates (25 *μ*L) from 10 pools were mixed into a super pool as described [27]. Total genomic DNA was extracted from each super pool using a modified sodium dodecyl sulfate (SDS)‐based DNA extraction [28]. Five hundred microliters of each super pool of tick homogenates were mixed with 300 *μ*L of DNA extraction buffer (50 mM Tris‐HCL, 25 mM sodium EDTA (pH 8.0), 25 mM NaCl, 10% SDS), and 10 *μ*L of Proteinase K. The samples were incubated in a 56°C water bath overnight with gentle inversions. The lysed samples were mixed with an equal volume of chloroform: isoamyl alcohol (24:1, *v*/*v*) and centrifuged at 10,000 rpm for 10 mins. The supernatant was transferred to another tube and mixed with an equal volume of chloroform: isoamyl alcohol and centrifuged at 10,000 rpm for 15 mins. The aqueous phase was precipitated with two volumes of ice‐cold absolute ethanol at −20°C for 2 h. The pellet of total nucleic acids was obtained by centrifugation at 13,000 rpm for 20 mins, washed with cold 70% ethanol, and resuspended in deionized water to a final 35 *μ*L volume. The integrity and quality of the extracted DNA were assessed by 1% (*w*/*v*) agarose gel electrophoresis in 1X TAE buffer.

The presence of *Anaplasma* and *Theileria* was screened by PCR using single‐plex genus‐specific primers (Table 1). The 10 *μ*L reaction PCR consisted of 2 *μ*L of extracted DNA samples, the GoTaq Green PCR master mix (Promega), forward and reverse primers for the specific reactions, and PCR water, according to the manufacturer′s instructions. Each assay of the different pathogens (*Anaplasma* spp. and *Theileria* spp.) had known positive and negative control (nuclease‐free water). *Theileria spp*. cycling conditions were (95°C (5 min), followed by 35 cycles of 94°C (1 min), 64°C (1 min), and 72°C (1 min); the last extension was 72°C (7 min). *Anaplasma* spp., cycling conditions were 95°C for 5 min; 35 cycles of 94°C for 45 s, 58°C for 30 s, and 72°C for 1 min; final extension at 72°C for 7 min. Amplicons were visualized using 1% agarose gel prestained with ethidium bromide.

**Table 1:** Genus‐specific primers used to identify *Theileria* and *Anaplasma* spp.

Target pathogen	Target gene	Primer name	Sequence (5 ^′^–3 ^′^)	Amplicon size (bp)	Reference
*Theileria* Spp.	18S rRNA	RLB F	GACACAGGGAG GTAGTGACAAG	450	[29]
		RLB R	CTAAGAATTTCA CCTCTGACAGT		
*Anaplasma* Spp.	16S rRNA	AnaplasmaJVF	CGGTGGAGCATG TGGTTTAATTC	300	[30]
		AnaplasmaJVR	CGRCGTTGCAAC CTATTGTAGTC		

### 2.4. Sanger Sequencing and Phylogenetic Analysis

Positive PCR products were sent to the University of Nairobi Institute of Tropical and Infectious Diseases (UNITID) laboratories for bidirectional Sanger sequencing (3 ^′^–5 ^′^ and 5 ^′^–3 ^′^) using the same primers as in PCR amplification in an ABI 3130 Genetic Analyzer system (Applied Biosystems). Sequences were analyzed in Geneious Prime v9.0.5, aligned via MUSCLE, and compared with NCBI/GenBank using BLAST [31]. Neighbor‐joining phylogenetic trees (bootstrap = 1000 replicates) were constructed [32] to determine the evolutionary relationships and geographic context. This involved comparing the sequences from this study with reference strains and previously published isolates to understand their genetic relatedness and potential transmission links.

## 3. Results

Overall, a total of *n* = 716 ticks were collected and identified to three genera and nine species. *Rhipicephalus e. evertsi* was the most predominant (*n* = 585, 81.7%) followed by *Rhipicephalus appendiculatus* (*n* = 24, 3.4%), *H. truncatum* (*n* = 17, 2.4%), *A. Gemma* (*n* = 15, 2.1%), *H. albiparmatum* (*n* = 9, 1.3%), *A. variegatum* (*n* = 6, 0.8%), *H. marginatum* (*n* = 4, 0.6%), *A. hebraeum* (*n* = 1, 0.1%), and *Rh. pulchellus* (*n* = 1, 0.1%). Unidentified nymphs constituted 7.5% of the sample (*n* = 54).

Tick species distribution varied across the sampled sites as follows: Osero 21 site exhibited the highest tick burden (*n* = 478). This area was dominated by *Rh. appendiculatus* (*n* = 436), whereas the Karera 6 site had the lowest tick burden, with only 16 ticks being collected (Table 2). *Rhipicephalus* species were the most widely distributed genus across all the sites, whereas *Amblyomma* and *Hyalomma* species were the least predominant across all the locations.

**Table 2:** Distribution of tick taxa and life stages across sampling sites.

Taxon	Gatune (*n* = 32; 4.5%)	Osero (*n* = 478; 66.8%)	Nachu (*n* = 28; 3.9%)	Riu Ndari (*n* = 58; 8.1%)	Karai bomboini (*n* = 74; 10.3%)	Karera 6 (*n* = 16; 2.2%)	Karera 8 (*n* = 30; 4.2%)	Grand total (*N* = 716)
***Amblyomma***								**22 (3.1%)**
*A. gemma*	–	14 (2.9%)	1 (3.6%)	–	–	–	–	15 (2.1%)
*A. hebraeum*	–	–	–	–	–	–	1 (3.3%)	1 (0.1%)
*A. variegatum*	4 (12.5%)	2 (0.4%)	–	–	–	–	–	6 (0.8%)

***Hyalomma***								**30 (4.2%)**
*H. albiparmatum*	2 (6.3%)	4 (0.8%)	–	2 (3.4%)	–	1 (6.3%)	–	9 (1.3%)
*H. marginatum*	1 (3.1%)	3 (0.6%)	–	–	–	–	–	4 (0.6%)
*H. truncatum*	2 (6.3%)	9 (1.9%)	3 (10.7%)	3 (5.2%)	–	–	–	17 (2.4%)

***Rhipicephalus***								**610 (85.2%)**
*Rh. appendiculatus*	2 (6.3%)	2 (0.4%)	3 (10.7%)	9 (15.5%)	5 (6.8%)	2 (12.5%)	1 (3.3%)	24 (3.4%)
*Rh. e. evertsi*	21 (65.6%)	436 (91.2%)	21 (75.0%)	13 (22.4%)	53 (71.6%)	13 (81.3%)	28 (93.3%)	585 (81.7%)
*Rh. pulchellus*	–	–	–	1 (1.7%)	–	–	–	1 (0.1%)

**Life stage**								**54 (7.5%)**
Nymphs	–	8 (1.7%)	–	30 (51.7%)	16 (21.6%)	–	–	54 (7.5%)

*Note:* Values represent the number of ticks collected at each site, with percentages in parentheses indicating the proportion of ticks within each site. Percentages in the grand total column represent the proportion of the overall collection (*N* = 716). “–” indicates zero counts. The bolded values are the total number of ticks that were identified for each of the genera.

Cattle hosted the highest species richness (*S* = 8), including the only observations of *A. variegatum* (*n* = 6) and *Rh. pulchellus* (*n* = 1) (Table 3). Cattle also accounted for all 54 nymphs collected in the study. Sheep had the highest total tick burden (*n* = 333), largely driven by a heavy infestation of *Rh. e. evertsi* (*n* = 308), which comprised 92.5% of the ticks found on this host. Goats exhibited the lowest tick burden (*n* = 68) and the lowest richness (*S* = 5). The overall Shannon Diversity Index (H ^′^) for the study area was 0.786, with a Pielou′s evenness (J ^′^) of 0.341. When disaggregated by animal host, cattle had the highest diversity (H ^′^ = 0.978) and relatively high evenness (J ^′^ = 0.47). In contrast, sheep showed the lowest diversity (*H* ^′^ = 0.372) and the lowest evenness (J ^′^ = 0.207), reflecting the extreme dominance of *Rh. e. evertsi* on this host. Goats maintained the highest evenness score (J ^′^ = 0.476), despite their lower total abundance.

**Table 3:** Biodiversity indices of tick species collected from different animal hosts in Kiambu County, Kenya. The table summarizes the total number of ticks collected (*N*), species richness (number of distinct tick species), Shannon Diversity Index (H ^′^), and Pielou′s evenness index (J ^′^) for cattle, goats, and sheep.

Animal host	Total ticks (*N*)	Species richness	Shanon index (H ^′^)	Pielou′s eveness (J ^′^)
**Cattle**	315	8	0.978	0.47
**Goat**	68	5	0.765	0.476
**Sheep**	333	6	0.372	0.207
**Total**	716	10	0.786	0.341

### 3.1. Pathogen Prevalence

PCR detected *T. parva* in 8/129 tick pools and *Anaplasma* spp. in 5/129 pools. Seven *T. parva*‐positive samples were localized to one site, with one additional sample originating from Karera 8, indicating broader distribution. All *T. parva*‐positive samples were detected in *Rh. e. evertsi* ticks. *Anaplasma* species were detected in *n* = 3 pools of *Rh. e. evertsi*, *n* = 1 pool of *A. variegatum*, and *n* = 1 pool of *H. truncatum* ticks. 16S rRNA sequencing of samples collected from this study identified *A. ovis* strain with 100% identity to global strains in ticks from cattle and sheep in Eastern Spain (GenBank ID: PP530070), South Africa (GenBank ID: OQ909443), and China (GenBank ID: OQ701064), indicating a widespread distribution of this pathogen. One *Anaplasma* sequence from sheep‐associated ticks showed 98.9% similarity to *A. ovis*, whereas another shared 99.1% identity with *A. bovis*, suggesting possible intraspecies genetic variation.

### 3.2. Genetic Variability

**Figure 2:**
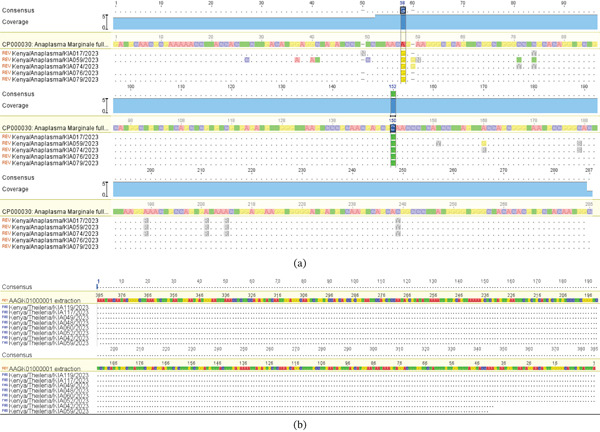
(a) Alignment of 16S rRNA gene sequences from *Anaplasma* samples against the *A. marginale* reference genome, highlighting two single nucleotide polymorphisms (SNPs; vertical bars) at positions 58 and 150. (b) Multiple sequence alignment of 16S rRNA gene sequences from Kenyan *Theileria* samples against the *T. parva* reference genome.

Alignment of *Anaplasma* spp. with the *A. marginale* reference genome (CP000030) revealed two single nucleotide polymorphisms (SNPs) (C → T at position 58, *p* = 3.2*E* − 39; A → G at position 152, *p* = 1.0*E* − 26) (Table 4, Figure 2a). *T. parva* sequences showed no polymorphisms in the 18S rRNA region compared with the *T. parva* Muguga strain (Figure 2b).

**Table 4:** *Anaplasma* spp. showed single nucleotide polymorphisms (SNPs) identified at positions 58 and 150 indicating the type of transition, the variant frequency, and the *p*‐statistic (*p* < 0.01).

Position	Change	Coverage	Polymorphism type	Variant frequency	Variant *p* value
58	A ‐> G	5	SNP (transition)	100.00%	3.2E‐39
150	G ‐> T	5	SNP (transition)	100.00%	1.0E‐26

### 3.3. Phylogenetic Analyses of *Anaplasma* and sequences

**Figure 3:**
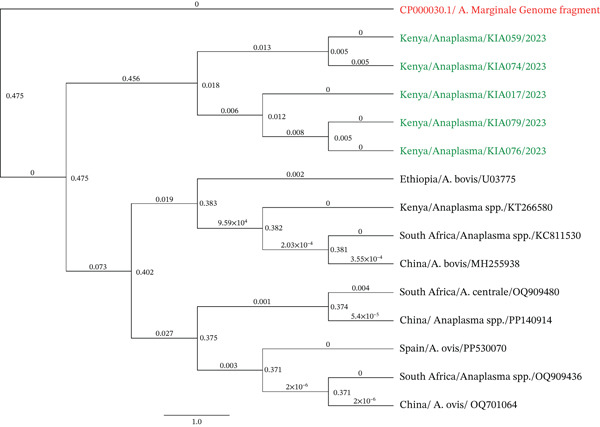
Maximum Likelihood phylogenetic tree of *Anaplasma* spp. based on 16S rRNA sequences. Bootstra*p* values (> 70%) from 1000 replicates are shown at the nodes. Study isolates (green) are compared with reference sequences from GenBank (red) and global isolates (black). Scale bar represents genetic distance.

The Kenyan *Anaplasma* samples formed a tight cluster (Figure 3) with the minimal branch lengths indicating a close genetic relationship within the group. This Kiambu clade grouped directly with the *A. marginale* reference sequence (CP000030.1), showing no genetic distance. The study isolates from Ethiopia, South Africa, and China clustered closely with the study isolates, demonstrating close genetic relatedness between the isolates.

**Figure 4:**
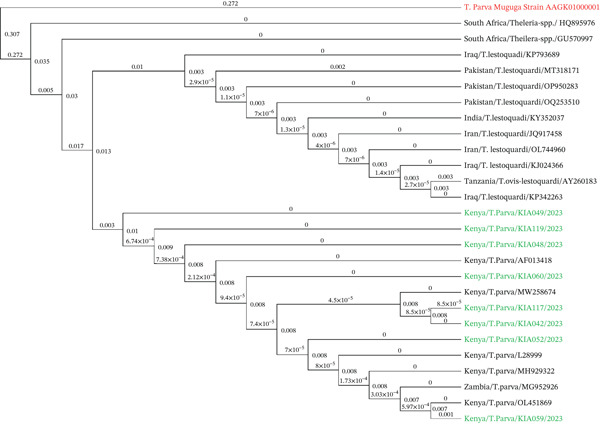
Phylogenetic tree of *T. parva* based on partial 18S rRNA gene sequences. The tree was inferred using the maximum likelihood method with 1000 bootstrap replicates to evaluate the robustness of clades. Bootstra*p* values ≥ 70% are displayed at key nodes. Study isolates from Kiambu County, Kenya are shown in green, the *T. parva* Muguga reference strain is in red, and additional global reference sequences (e.g., Uganda, Mexico, Zimbabwe) are shown in black. Branch lengths correspond to genetic distances based on the number of nucleotide substitutions per site.

The *T. parva* samples from this study were clustered on various branches of the tree. This clustering between samples in the study suggests a high degree of genetic similarity among these samples (Figure 4). The low genetic distances between the samples suggest closer relationships [31] between the samples in this study and previously characterized samples from Shimba Hills (OL451869), Maasai Mara (MH929322), Mexico (MG952926), Uganda (L28999), Zimbabwe (AF013418), and Coastal Kenya (MW258674). Furthermore, the presence of the samples used in this study across various branches of the tree suggests a diverse range of *T. parva* strains.

## 4. Discussion

Tick‐borne pathogens are of public health and veterinary importance. Livestock productivity affects several sectors including food security and rural economies. The detection of *Theileria* and *Anaplasma* in Kiambu County highlights the continued burden of TBDs on livestock health.

This study demonstrates the dominance of *Rhipicephalus* species, particularly *Rh. e. evertsi* and *Rh. appendiculatus*, across the surveyed sites and hosts. *Rh. e. evertsi* was the most predominant tick species at 81.7%, aligning with its role as the key vector for *T. parva* in Africa [33, 34]. The presence of *Rh. e. evertsi* across the three host types (cattle, sheep and goats) shows its opportunistic feeding habits, its successful adaptation in the study area and importance in disease transmission across a wide range of hosts. The significant variation in tick abundance and species composition between sites for example, the extreme abundance of *Rh. e. evertsi* at Osero 21 compared with Nachu reflects differences in local environmental conditions. The local environmental conditions include vegetation, humidity, temperature, host density, movement patterns, and potential variations in acaricide application or control practices between locations. The high nymph burden on cattle likely results from a combination of tick life cycle strategies, especially two‐host strategies in *Rh. e. evertsi*, host‐specific or opportunistic attachment behavior. In addition, environmental and management factors that make cattle the principal, or sometimes exclusive, suitable host for nymphal stages in the study area.

The tick population in Kiambu County exhibited low overall diversity and strong dominance by *Rh. e. evertsi*, as reflected by a low Shannon Diversity Index (*H* ^′^ = 0.786) and evenness (*J* ^′^ = 0.341). Cattle, despite hosting fewer ticks than sheep, supported the greatest species richness (*S* = 8) with the most nymphs, indicating their role as key biodiversity reservoirs. In contrast, sheep showed the lowest evenness (*J* ^′^ = 0.207), suggesting a highly specialized association with *Rh. e. evertsi*, which may act as a primary amplifier host for this vector species. Goats exhibited moderate evenness (*J* ^′^ = 0.476) despite low tick abundance, implying a lesser role in tick proliferation, possibly due to behavioral or physiological resistance factors.

The high abundance of *Rh. appendiculatus* and *Rh. e. evertsi* is of significant veterinary and public health concern. *Rh. appendiculatus* is the primary vector for *T. parva*, causing East Coast fever in cattle. *Rh. e. evertsi* on the other hand can transmit zoonotic pathogens, including *Theileria*, *Babesia*, *Anaplasma*, and *Rickettsia* species that cause spotted fever group rickettsioses in humans. Additionally, its bites may induce allergic and toxic reactions, posing direct health risks. The presence of *Amblyomma* species (*A. variegatum*, *A. gemma*, *A. hebraeum*) introduces the risk of diseases like heartwater (*Ehrlichia ruminantium*) and potentially rickettsioses. *Hyalomma* species are vectors for diseases like Crimean‐Congo hemorrhagic fever (CCHF) virus and theileriosis. CCHF virus has been detected in several tick species in Kenya including *Hyalomma rufipes* and *H. truncatum [* *35* *]*, as well as CCHF antibodies in humans in pastoral communities in Kenya [36].

The *T. parva* prevalence (7%) was lower than in pastoral regions like Machakos (66%) [37], likely due to zero‐grazing practices in Kiambu [38] and fewer *Rh. appendiculatus* ticks, its primary vector [39].

*A. ovis* was detected in ticks from cattle and sheep, with sequences showing 100% identity to global strains from Spain, South Africa, and China (GenBank IDs: PP530070, OQ909443, OQ701064), confirming its widespread distribution. These findings are in line with previous studies in Germany [40] and Sardinia [41] that demonstrate the widespread occurrence of *A. phagocytophilum* and other Anaplasma species in sheep and cattle. This emphasizes the need for continuous surveillance and control measures. Notably, sheep‐derived ticks exhibited slight genetic divergence (98.9%–99.1% homology to *A. ovis* and *A. bovis*), suggesting intraspecies variation or cocirculation of related species. Previously, *A. ovis* was detected in the serum of goats using the ELISA technique in various parts of Kenya [42], whereas *A. bovis* was detected in cattle in the wildlife–livestock interface in Lambwe Valley, western Kenya [19]. These findings underscore the importance of veterinary interventions to mitigate economic losses resulting from anemia, reduced fertility, and mortality in livestock [43].

A sequence derived from a male *Rh. e. evertsi* tick from Karai Bomboini showed 99.4% homology to *A. ovis*, implicating this species in regional transmission in line with previous studies in Maasai Mara National Reserve [44] where *A. ovis* was detected in questing *Rh. evertsi ticks.*

Genetic variations in tick‐borne pathogens, arising from mutations or recombination events, can significantly impact their pathogenicity, transmission dynamics, and geographic distribution. This study highlights such variations in *Anaplasma* and *T. parva* populations. SNPs (C → T at position 58; A → G at position 152) within *Anaplasma* sequences may indicate local adaptation or selection pressures, possibly driven by host immune response, environmental conditions, or vector‐specific factors. These mutations could influence pathogen virulence, transmission efficiency, or diagnostic detectability and warrant further investigation.

Phylogenetically, Kenyan *Anaplasma* strains clustered closely with Ethiopian and South African isolates, indicating shared ancestry or transmission routes [45, 46], but distantly with those from China and Spain, suggesting a greater degree of evolutionary divergence. This regional clustering contrasts with divergent strains from Europe/Asia, likely due to geographic isolation and host‐environment dynamics.

The 18S rRNA gene is often used in phylogenetic studies due to its relatively slow rate of evolution, making it reliable for identifying species and tracing broader evolutionary relationships [47]. For *T. parva*, the conserved 18S rRNA sequences shared a high degree of similarity to the well‐characterized *T. parva* Muguga reference strain. When compared with other *T. parva* isolates reported globally, the Kenyan isolates clustered closely with isolates from Uganda (GenBank accession L28999) and Mexico (GenBank accession MG952926), showing possible interconnectedness among the isolates and the existence of transmission links for *T. parva*.

This study was limited by its sample size and geographical scope, which may not fully capture the diversity of tick‐borne pathogens in Kenya. Additionally, sequencing of only PCR positive samples may underrepresent the true genetic diversity. Future work should include whole‐genome sequencing and broader ecological sampling to better understand pathogen dynamics and adaptation.

## 5. Conclusion

This study reports that cattle play a critical role in maintaining the diversity of the tick community, hosting the widest variety of species and life stages. Conversely, sheep are the most significant hosts for proliferation of *Rh*. *e. evertsi*. The dominance of *Rh. e. evertsi* ticks in Kiambu County, Kenya, and their role in transmitting *T. parva*, the causative factor for Theileriosis, poses risks to livestock health. The distribution of *T. parva* among these tick species collected predominantly from one site suggests localized disease hotspots. Molecular analysis identified SNPs (C → T and A → G) in *Anaplasma* spp. sequences. This suggested localized genetic variation. Phylogenetic analysis revealed that Kenyan Anaplasma strains clustered closely with other African isolates, such as those from Ethiopia and South Africa, indicating regional transmission and genetic continuity within the continent.

These findings emphasize the need for targeted surveillance of *T. parva* and *Anaplasma* spp. to detect emerging variants, livestock management strategies (e.g., acaricide use, zero‐grazing reinforcement) to reduce tick‐borne disease burdens, and Genetic characterization of pathogens to inform control policies and mitigate economic losses.

We recommend implementing integrated vector management strategies, including targeted acaricide application, environmental modifications, and livestock movement control, to reduce tick burdens. Additionally, ongoing molecular surveillance using high‐throughput sequencing and SNP profiling should be prioritized to detect emerging strains, monitor resistance patterns, and guide region‐specific control measures.

## Author Contributions

Peter Gichuki: conceptualization, investigation, writing—original draft, writing—review and Editing. Caroline Wasonga: conceptualization, resources, writing—review and editing, Supervision, funding acquisition. Christine Adhiambo: writing—review and editing, supervision. Joel Lutomiah: resources, writing—review and editing, supervision, funding acquisition.

## Funding

This study was supported by International Veterinary Vaccinology Network.

## Ethics Statement

This study was conducted in accordance with the ethical guidelines and regulations governing research on vector‐borne diseases. The collection of ticks was approved under the Scientific and Ethics Review Unit (SERU) Approval Number KEMRI/SERU/CVR/004/3926. All procedures adhered to institutional and national ethical standards to ensure responsible handling of biological samples. No human participants were involved in this study, and no ethical concerns regarding human subjects arose.

## Conflicts of Interest

The authors declare no conflicts of interest.

## Supporting information


**Supporting Information** Additional supporting information can be found online in the Supporting Information section. The table provides raw metadata for all individual tick pools used in this study. Each row represents a single pool that was processed for analysis. The names and unique identifiers for all geographic sites are also provided.

## Data Availability

The data that supports the findings of this study are available in the supporting information of this article.
