# Correction to “Genetic Diversity of *Theileria parva* and *Anaplasma* spp. Isolated From Ticks Collected From Kiambu County, Kenya”

**DOI:** 10.1155/bmri/9864241

**Published:** 2026-10-23

P. Gichuki, C. Wasonga, C. Adhiambo, J. Lutomiah, “Genetic Diversity of *Theileria parva* and *Anaplasma* spp. Isolated From Ticks Collected From Kiambu County, Kenya,” *BioMed Research International*, 2026, 5577637, https://doi.org/10.1155/bmri/5577637

In the article, Zambia was incorrectly referred to as Mexico in multiple instances. All references to Mexico should instead read Zambia.

We apologize for these errors.

